# A novel mutation tolerant padlock probe design for multiplexed detection of hypervariable RNA viruses

**DOI:** 10.1038/s41598-019-39854-3

**Published:** 2019-02-27

**Authors:** Sibel Ciftci, Felix Neumann, Iván Hernández-Neuta, Mikhayil Hakhverdyan, Ádám Bálint, David Herthnek, Narayanan Madaboosi, Mats Nilsson

**Affiliations:** 1 Science for Life Laboratory, Department of Biochemistry and Biophysics, Stockholm University, SE- 171 65, Solna, Sweden; 2 Department of Microbiology, National Veterinary Institute (SVA), Ulls väg 2B, SE 751 89, Uppsala, Sweden; 3 National Food Chain Safety Office Veterinary Diagnostic Directorate (NEBIH), Tábornok Str. 2, H-1149 Budapest, Hungary

## Abstract

The establishment of a robust detection platform for RNA viruses still remains a challenge in molecular diagnostics due to their high mutation rates. Newcastle disease virus (NDV) is one such RNA avian virus with a hypervariable genome and multiple genotypes. Classical approaches like virus isolation, serology, immunoassays and RT-PCR are cumbersome, and limited in terms of specificity and sensitivity. Padlock probes (PLPs) are known for allowing the detection of multiple nucleic acid targets with high specificity, and in combination with Rolling circle amplification (RCA) have permitted the development of versatile pathogen detection assays. In this work, we aimed to detect hypervariable viruses by developing a novel PLP design strategy capable of tolerating mutations while preserving high specificity by targeting several moderately conserved regions and using degenerate bases. For this, we designed nine padlock probes based on the alignment of 335 sequences covering both Class I and II NDV. Our PLP design showed high coverage and specificity for the detection of eight out of ten reported genotypes of Class II NDV field isolated strains, yielding a detection limit of less than ten copies of viral RNA. Further taking advantage of the multiplex capability of PLPs, we successfully extended the assay for the simultaneous detection of three poultry RNA viruses (NDV, IBV and AIV) and combined it with a paper based microfluidic enrichment read-out for digital quantification. In summary, our novel PLP design addresses the current issue of tolerating mutations of highly emerging virus strains with high sensitivity and specificity.

## Introduction

The ever-increasing numbers of viruses are posing a great threat by causing unexpected health issues, including epidemics, in both humans and animals. Studies highlight that approximately one new virus strain emerges each year, and the capacity to identify and control emerging diseases due to these new pathogen strains remains challenging^1^. In particular, RNA viruses are prone to rapid evolution, with single-stranded RNA viruses mutating faster than double-stranded ones^2^, as a consequence of the lack of proofreading activity of their RNA polymerases^3^, and their sensitivity to chemicals and oxidative deamination. In order to combat the issue of handling such emerging strains, a WHO/OIE panel has called for timely and responsive disease surveillance systems for both, animal and human populations^4^, also throwing light on currently available poor information related to viral subpopulations or quasispecies, which in turn hinders their accurate diagnosis.

This problem is prominent among poultry viruses, where a high number of strains pose a serious threat to both, meat and egg production, as well as risk of turning into human epidemics. Examples of such high virulent viruses include Newcastle Disease Virus (NDV), Avian Leukosis Virus SubgroupJ, Infectious Bursal Disease Virus (IBDV), Infectious Bronchitis Virus (IBV), Avian Influenza Virus (AIV) and Infectious Laryngotracheitis Virus (ILTV). Among these, NDV poses a major threat to poultry industry and also humans, when in a transmissible form, causing serious economic losses worldwide^5^. In addition, Exotic Newcastle disease is a very deadly form that causes sudden dead of birds with no prominent symptoms^6^. NDV is a non-segmented negative sense RNA virus containing a 15 kb genome, belonging to the *Paramyxoviridae* family^7^. Of all the six genes identified within the genome, F- and L-genes are relevant for the grouping of NDV^8^. The L-gene coding for RNA-dependent RNA polymerase shares fundamental attributes for viral replication and transcription^9^, thus serving as candidates for targeting hypervariable viruses owing to their relative conservancy. The known NDV strains are grouped under two main classes (Class I and II), comprising of 19 established genotypes with subpopulations and quasispecies, associated with spatio-temporal and host species delineations^10,11^. Molecular pathotyping of diverse strains which is usually performed using F gene Reverse transcription – PCR (RT-PCR) has helped in understanding and classifying NDV strains based on their virulence into one of the three categories, lentogenic, mesogenic and velogenic^12,13^. The most widely used techniques for detection and typing of NDV strains include isolation of virus and immunohistochemistry, as well as RT-PCR, gene sequencing and microarrays^14,15^. All these methods have strengths and weaknesses in terms of sensitivity and specificity, assay integration, automation and field-based diagnosis.

Padlock probes (PLPs) in combination with Rolling Circle Amplification (RCA), an isothermal nucleic acid amplification method, comprise a robust molecular technique that has been used for molecular detection and typing of different pathogens^16–20^. A PLP is a single-stranded linear DNA oligonucleotide that contains 15–20 nt long target complementary arms and 40–50 nt non-hybridizing backbone. Upon binding of the two PLP arms in a juxtaposed position to its target, a circle is formed which can be linked enzymatically through DNA ligation^21^. The use of PLPs provides a high level of specificity since complete hybridization of both arms and a perfect match at the ligation site are required^21^. The circularized probes are amplified by target-primed RCA using *phi29* DNA (*Φ29*) polymerase, which generates approximately 900 copies/hour of the circularized PLP^22^. These RCA products (RCPs) are submicron-sized single-stranded DNA concatemers that can be digitally quantified in solution^23,24^ after fluorescence labelling, thus offering distinct advantages over other isothermal amplification methods. For more sensitive applications, RCPs can be further amplified by subsequent rounds of RCA (Circle-to-circle amplification, C2CA) by monomerizing RCPs with a  restriction enzyme and re-ligating the resulting monomers^23^. While assays for detection of viruses such as Crimean-Congo hemorrhagic fever virus (CCHFV), rotavirus, and human influenza (type A and B) have already been demonstrated using PLPs and RCA^16,17,25^, such assays either target several sites using single PLPs each^25^ or using multiple PLPs to a single target site^16,17^, thereby overlooking the problem of mutation tolerance for detecting emerging strains.

We herein present a novel PLP design strategy with a C2CA-based assay to tackle this issue of mutation tolerance for NDV detection. For this, we designed probes targeting different moderately conserved sites in the L-gene of NDV and degenerate bases in the probe arms, aiming to be able to detect a wide number of field isolates with high sensitivity, while maintaining high stringency provided by the PLP ligation reaction. Furthermore, we fully exploited the use of PLPs extending the assay to simultaneously detect NDV, IBV and AIV. Therefore, we extended the scope of a recently published simple and rapid microfluidic enrichment strategy to quantify RCPs in a multiplexed way with enhanced sensitivity^26^. We integrated a mutation-tolerant PLP design and the membrane-based detection tool to demonstrate a sensitive PLP-based C2CA assay for the detection of highly mutating viral strains.

## Materials and Methods

### Virus propagation and isolates

A velogenic NDV genotype VII strain 14036/2006 and a mesogenic pigeon paramyxovirus type 1 (PPMV-1) 202/2016 isolated from severe clinical cases and reference NDV strain Herts/33, along with other NDV strains and avian viruses in this study, were propagated in embryonated chicken eggs according to standard protocols. All methods were carried out in accordance with relevant guidelines and regulations of OIE from the Manual of Diagnostic Tests and Vaccines for Terrestrial Animals 2018 (Chapter 2.3.14 Newcastle Disease). All experimental protocols were approved by the NFSCO-DVD (National Food Chain Safety Office – Directorate for Veterinary Diagnostics) animal experiment committee.

Influenza type B/Stockholm/5/2014 (Victoria) strain was grown in Madin-Darby canine kidney cells (MDCK). Supernatants were obtained from cell culture as described previously^20^. Details of viral strains used in this study are summarized in SI, ST4.

### Primer and padlock probe design

Sequences of 335 different NDV strains, with known NCBI accession numbers, were obtained from the Swedish National Veterinary Institute (SVA), and aligned using Geneious 6.1.8. (Biomatters Ltd.). Out of the 335 sequences, 21 sequences covered Class I NDV, and the remaining 314 sequences covered the reported genotypes from Class II NDV, thus representing both A and B groups in the study. To confirm the specificity of the aligned sequences, BLASTn was performed against the selected target regions, for NDV groups A and B represented in the generated phylogenetic tree. The phylogenetic tree was constructed using Geneious Tree builder with the following chosen settings: Tamura-Nei as genetic distance model, neighbour-joining as tree build method, with no outgrouping. For these regions, 9 primers for reverse transcription (RT) and 9 PLP sequences (Table 1) (with the inclusion of degenerate bases, generating 21 unique PLPs) were designed, along with their respective synthetic targets (SI, ST1), covering 3 subgroups in group A and 6 in group B. For IBV, nucleocapsid gene sequences of 405 strains were aligned using Geneious. A phylogenetic tree was constructed and the selected 7 groups were arbitrarily annotated as follows; A, B, C, D, E, F. Similar parameters and rationale used for NDV PLP design were also applied for IBV, however more sequence grouping and less wobble usage were considered in this case (SI, Fig. S1). Finally, 11 PLPs in total with 15 unique oligonucleotides were generated in order to cover all the aligned IBV sequences (SI, ST2).

**Table 1:** Table of oligonucleotides used in this study.

Name	5′ modification	Sequences (5′ to 3′ direction)
NDV.A1_PLP	Phosphate	CAATWCCCCCTCTCGCAGTGTATGCAGCTCCTCAGTAATAGTGTCTTACGACGCAACTTCACCGAATGATTCTGGCAAAGCCCCT
NDV.A2_PLP	Phosphate	YCTGATAGTCTCTCTGTCTTGTGTATGCAGCTCCTCAGTAATAGTGTCTTACGACGCAACTTCACCGAATGATAGATGAAGAAAGTATCTGA
NDV.A3_PLP	Phosphate	ATGGAGACACTCGATATAGTGTATGCAGCTCCTCAGTAATAGTGTCTTACGACGCAACTTCACCGAATGAAGTCATTTGATATATGGACAT
NDV.B1_PLP	Phosphate	TGACCCCATTTCCCTGGTGTATGCAGCTCCTCAGTAATAGTGTCTTACGACGCAACTTCACCGAATGAAAGGATATGCTATCYTGAA
NDV.B2_PLP	Phosphate	RTTAAGACAATACTTTTGCAGTGTATGCAGCTCCTCAGTAATAGTGTCTTACGACGCAACTTCACCGAATGATTGACTGTCTGATATCTCCA
NDV.B3_PLP	Phosphate	ATTACAGTGTTTTCACGTGTATGCAGCTCCTCAGTAATAGTGTCTTACGACGCAACTTCACCGAATGAGATGCAATGTTGGCACARGAC
NDV.B4_PLP	Phosphate	TTCTRTTCCGGGCATAATCTGTGTATGCAGCTCCTCAGTAATAGTGTCTTACGACGCAACTTCACCGAATGACCTGTCAAAGGTGACCAGC
NDV.B5_PLP	Phosphate	RGATATGTGAATGTAAGGTGGTGTATGCAGCTCCTCAGTAATAGTGTCTTACGACGCAACTTCACCGAATGAAATAGCCTTTGRGAATCATT
NDV.B6_PLP	Phosphate	TTTTTGTTGAGCACGAGTCAGTGTATGCAGCTCCTCAGTAATAGTGTCTTACGACGCAACTTCACCGAATGACCTATGGTTTTCATRTASAA
NDV.A1 primer	Biotin	GTCCCGAATGACGACATATA
NDV.A2 primer	Biotin	CATGTCAATCATCTGATAGG
NDV.A3 primer	Biotin	ACTGCAGGGAATCTCCAACA
NDV.B1 primer	Biotin	AAGAGTATGYTRGCRATGAG
NDV.B2 primer	Biotin	AAGAGTATGYTRGCRATGAG
NDV.B3 primer	Biotin	GTCCTYAAAAAYTCATCTAA
NDV.B4 primer	Biotin	TYGCRCATGCYATCATGG
NDV.B5 primer	Biotin	YACRGCTGGGAATCTYCAACA
NDV.B6 primer	Biotin	TVGGDATTACYAAACTCAAAGA
Restriction oligo		GTGTATGCAGCTCCTCAGTA
Detection oligo	Cy3	TTTTTGTAAGACACTATTACTGAGG
Detection oligo*	Cy3	TTTTTTCATTCGGTGAAGTTGCGTC
Detection oligo*	Cy5	TTTTTTATCTCAGCACACGGGACAG
Detection oligo*	Alexa750N	TTTTTACAGGCAGGCAATCGTTGTA

W: A or T Y: C or T R: A or G S: C or G V: A or C or G D: A or G or T.

*Detection oligonucleotides used in multiplexing assay.

PLP cocktail for AIV was designed for the most conserved region in 6,683 sequences of the Matrix gene. A remarkably conserved region was identified between nucleotide 193 and 233 of the gene. Wobble was used for every mismatch base encountered near the ligation site. Therefore, the resulted PLP covered 99.5% coverage of all the aligned AIV strains (SI, ST2).

Sequences for NDV, IBV and AIV used in this study were obtained from NCBI, then short sequences were filtered out and the fragments that belong to the gene of interest were extracted and saved as a separate fasta file using CLC Genomics Workbench (CLCBio-Qiagen, v.7.5.2) software. Extracted sequences were aligned using CLC Genomics Workbench. Finally, duplicate sequences were removed using Jalview (Java Alignment Viewer, v.2.7;) software (Waterhouse, A.M. *et al*.^27^) which resulted in 335 sequences of NDV, 405 sequences of IBV and 6683 sequences of AIV^27^.

All oligonucleotides were obtained from Integrated DNA Technologies (Coralville, USA).

### RNA extraction and reverse transcription

**Figure 1:**
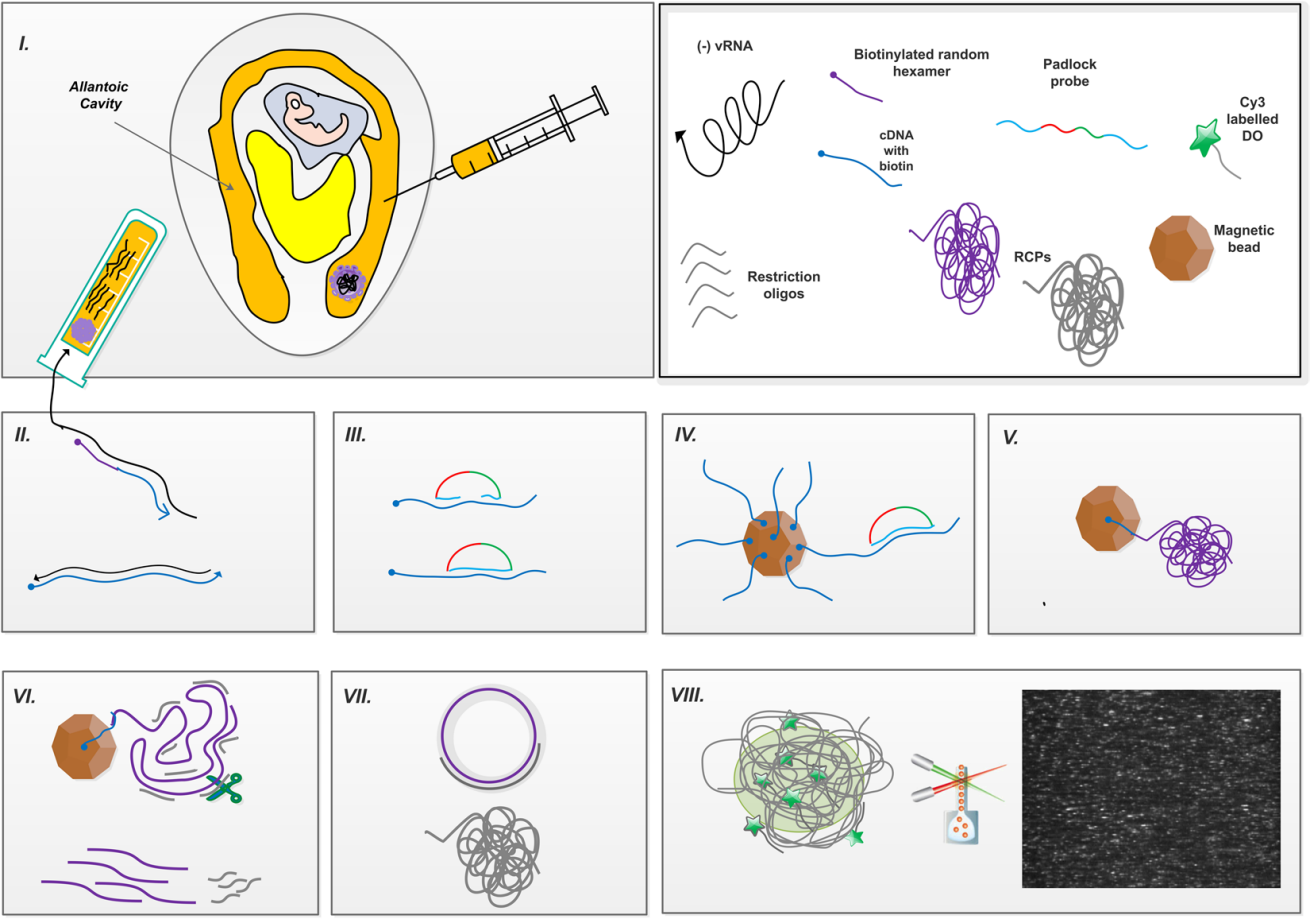
A schematic summary of the RT-C2CA assay representing the key steps (**I**). Egg inoculation via allantoic fluid route and virus RNA isolation (**II)**. Reverse transcription (RT) using biotinylated primers (**III)**. Hybridization of padlock probe (PLP) to the target, followed by ligation (**IV)**. Capture of ligated products on magnetic beads; (**V**). Rolling circle amplification (RCA) of captured molecules (**VI**). Monomerization of RCA products (RCPs) (**VII**). Ligation of monomerized fragments and second RCA (**VIII**). Fluorescence labelling of RCPs, followed by digital quantification using amplified single molecule detection (ASMD) – the inset shows an output image from the instrument, where individual RCPs become visible as single bright dots (in white, compared to the dark background) during analysis. Arrow indicates the polarity of the sequence from 5′ end to 3′ end.

RNA was extracted from the allantoic fluids of the infected embryonated eggs (Fig. 1. I) using the QIAamp Viral RNA Mini Kit as per the manufacturer’s protocol (Qiagen, Hilden, Germany). Influenza type B RNA was extracted with a MagNa Pure 96 instrument and MagNa Pure 96 DNA and Viral RNA Large Volume Kit (Roche).

The RNA samples were reverse transcribed applying the Superscript III First-strand Synthesis System (Invitrogen, Carlsbad, USA) with 2 µM gene-specific and 100 nM random primers in different combinations with RNaseH treatment (Blirt S. A., Gdansk, Poland) (Fig. 1. II).

NDV RNAs were reverse transcribed using biotinylated gene specific and/or random decamers primer, while IBV, IBDV, ILTV, AIV and influenza B cDNAs were made using only biotinylated random decamers primer.

### Circle-to-circle amplification

Phosphorylated PLPs were hybridized and ligated onto their targets by incubation in a mixture containing 10 μL of sample cDNA (or a chosen concentration of the synthetic target), 100 nM of each PLP, 0.2 μg/μL BSA (Sigma-Aldrich, Darmstadt, Germany), 0.125 U/μL Ampligase in Ampligase reaction buffer (Nordic Biolabs AB, Täby, Sweden) in a total volume of 20 µL, at 60 °C for 5 min (Fig. 1. III). Prior to use, SA-functionalized Dynabeads® T1 (10 μg/μL; Life Technologies, Oslo, Norway) were washed thrice with wash buffer (WB) containing 10 mM Tris-HCl (pH 7.5), 5 mM EDTA, 0.1 M NaCl and, 0.1% Tween-20 (Karolinska Institute Substrat, Stockholm, Sweden). The ligation mix was incubated with 5 μL of beads per reaction on rotation for 10 min at room temperature (Fig. 1. IV). Afterwards, the beads were washed once with WB to remove the unligated PLPs. To amplify the ligated circles, RCA mix containing 0.2 μg/μL BSA, 125 μM dNTPs (Blirt S. A., Gdansk, Poland, 200 mU/μL *Φ29* polymerase in 1x *Φ29* polymerase buffer (Monserate Biotechnology Group, San Diego, USA) was added and polymerization occurred at 37 °C for one hour, terminated at 65 °C for 2 min (Fig. 1. V). Using a magnet, the beads were collected and the supernatant was discarded. To monomerize the RCPs on beads, the digestion mix containing 0.2 μg/μL BSA, 120 mU/μL *Alu*I (New England Biolabs, Stockholm, Sweden) and 120 nM restriction oligonucleotide in 1x *Φ29* buffer was added and incubated for 5 min at 37 °C, followed by enzyme inactivation at 65 °C for 2 min (Fig. 1. VI). Monomers were ligated and a second round of RCA was performed in a mixture of 0.2 μg/μL BSA, 0.68 mM ATP (Blirt S. A., Gdansk, Poland), 125 μM dNTPs, 200 mU/μL *Φ29* polymerase and 0.02 U/μL T4 DNA ligase (Blirt S. A., Gdansk, Poland) in 1x *Φ29* buffer for one hour at 37 °C, followed by heat inactivation at 65 °C for 2 min (Fig. 1. VII).

### Quantification of Rolling Circle Amplification Products

The generated RCPs were labelled using detection oligonucleotides tagged with Cy3, Cy5 or Cy7 fluorophores (Table 1), 5 nM, in a 2X buffer mix containing 40 mM Tris-HCl (pH 8.0), 40 mM EDTA, 2.8 M NaCl and 0.2% Tween-20 in water. These labelled RCPs were quantified by amplified single-molecule detection (ASMD)^24^ using the dedicated instrument Aquila 400 (Qlinea AB, Sweden) (Fig. 1. VIII). The measurement average of technical duplicates was obtained, and the standard deviation was calculated.

For the microfluidic enrichment, a custom-made disposable microfluidic chip was used (Aline, USA)^26^. The chip was connected to a syringe pump through 1/32″ polytetrafluoroethylene (PTFE) Teflon tubing. The tubing was washed thrice with 20 µL of 0.05% sodium hypochlorite (Karolinska Institute Substrat, Stockholm, Sweden) and subsequently flushed with 40 µL of PBS buffer. After cleaning, the tubing was loaded with 20 µL of RCP sample and connected to the microfluidic enrichment chip via a short Tygon tubing connection. The liquid was brought close to the nitrocellulose membrane at a flow rate of 100 µL/min. Subsequently, the flow rate was decreased to 5 µL/min to enrich the RCPs on the membrane. Prior to imaging, the bottom protecting layer of the chip was removed. The embedded membrane had an active surface diameter of 1.5 mm, which corresponds approximately to the field of view of a 10x microscope objective.

### Image acquisition and processing

For fluorescent imaging, 15 focal plane images were acquired using Axioplan 2 epifluoresence microscope (Zeiss) under a 10x objective, orthogonally merged in Zen software (Zeiss) and subsequently analyzed for RCP signals using the CellProfiler software^28^. For image analysis, a CellProfiler pipeline was used containing image enhancement and manual thresholding for each fluorescence channel, as described elsewhere^26^.

## Results

### The rationale for padlock probe design

The current study includes a strategic design for molecular detection of rapidly evolving virus strains using PLPs and C2CA with NDV as a model. The PLPs were designed to be specific for the targeted gene regions, while at the same time being tolerant to mutations occurring with high frequency among virus strains. This was achieved by introducing degenerated nucleotide bases in the probe arm sequences, and also by targeting several sites within the L-gene.

**Figure 2:**
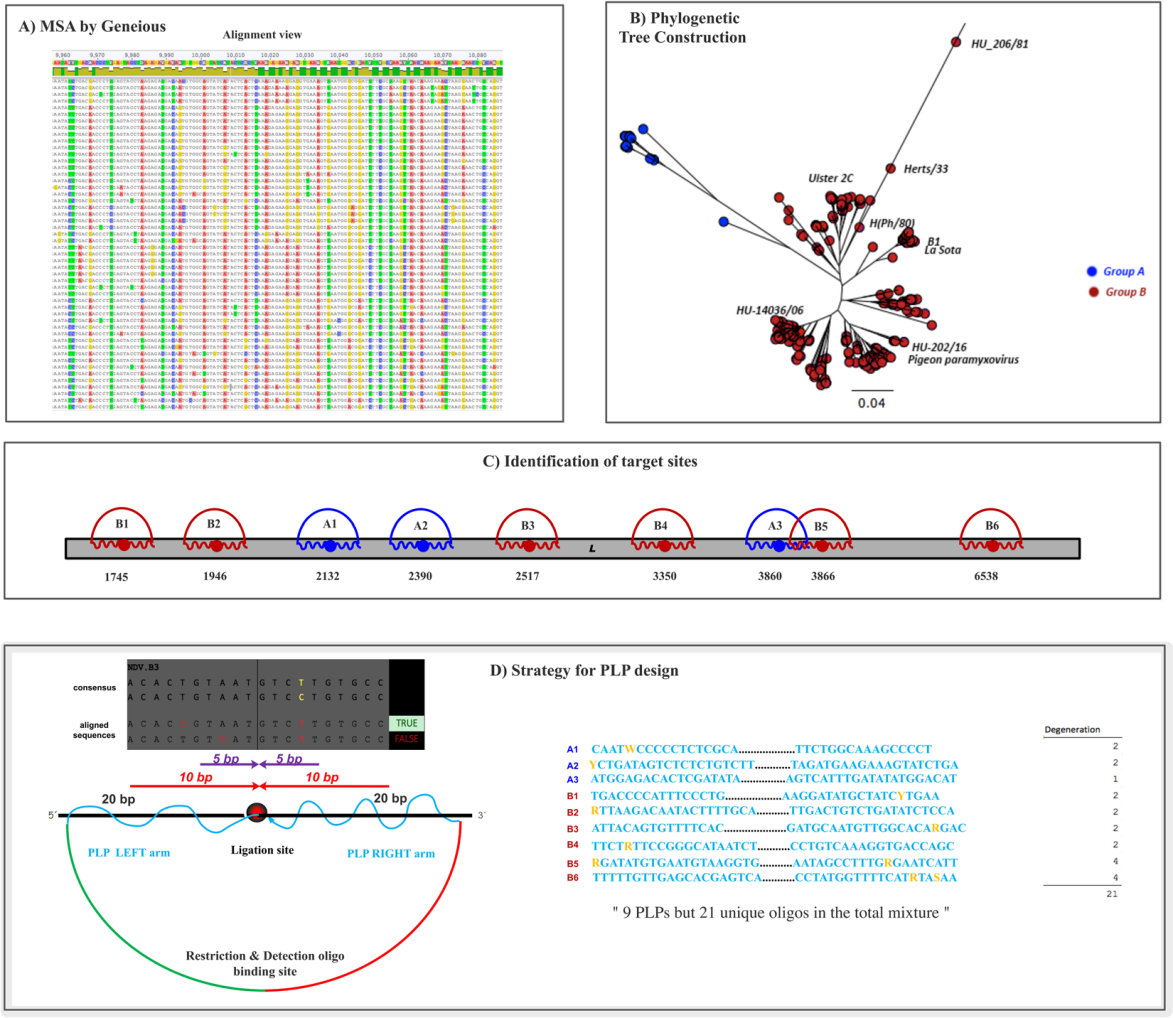
A schematic representation of PLP design strategy: (**A**). Multiple sequence alignment (MSA) of 335 NDV L-gene sequences using Geneious version 6.1.8, where the disagreements to the consensus sequences are highlighted in different colors; (**B**). Construction of unrooted phylogenetic tree using Neighbour-Joining method, showing the two groups **A** and **B** and the respective haplotypes; (**C**). Location of chosen target sites for PLP design, along the length of L-gene; (**D**). Extracted regions of L-gene sequences from the alignment view, representing the scoring for mismatches in the individual sequences relative to the consensus; seen below is the design of mutation-tolerant PLPs; seen on the right is the list of generated PLPs showing the inclusion of Wobbles.

Targeted regions were selected by aligning 335 NDV L-gene cRNA sequences (SI Table S2) using Geneious software (Fig. 2A). We constructed a phylogenetic tree based on this alignment as a guide to arbitrarily group the strains based on conservancy (Fig. 2B). The phylogenetic tree resulted in two large groups, which we named as A and B, together consisting of 9 subgroups (A1-3, B1-6). The sequences of individual subgroups were extracted in a separate analysis file (SI, Fig. S2) that was used to identify circa 40 bp relatively conserved regions as binding sites for individual PLPs (Fig. 2C). The extracted sequences, corresponded to 9 regions between 1,745 and 6,538 nt of the L-gene. We obtained the consensus sequences of each selected region and the conservancy score for each base was calculated. In order to cover possible mismatches in the PLP binding site, we took into account the fingerprint of the *Thermus thermophilus* DNA ligase, which ligates substrates extending 10 bp away on either side from the nick^29^ and being less tolerant to mismatches on the 3′ end than on the 5′ end of the nick site^30^. The ligation sites were positioned in such a way that the 3′ end included the most conserved regions of the target. No mismatches were allowed +/−5 bp from the nick site and any encountered mismatches in the individual aligned strain sequences to that of the consensus and wobbles were added to the probe arms when the variation was greater than 10% (Fig. 2D).

The final set of probes comprised of 9 PLPs with a mixture of 21 unique PLP sequences. By using this strategy, 90% of all the aligned sequences could be covered at a chosen target site for a given subgroup. By this way, instead of covering all variants at a single target site, as previously reported, we aim at covering most of the variants at several target sites.

### Assay design validation

To amplify ligated PLPs, we performed a C2CA-based assay, comprising of the following steps: RNA extraction from virus particles, reverse transcription, PLP hybridization and ligation performed on magnetic beads, two rounds of RCA and detection by ASMD (Fig. 1). The PLP efficiency and the C2CA conditions were optimized on synthetic targets designed to mimic the consensus sequences of the 9 subgroups.

**Figure 3:**
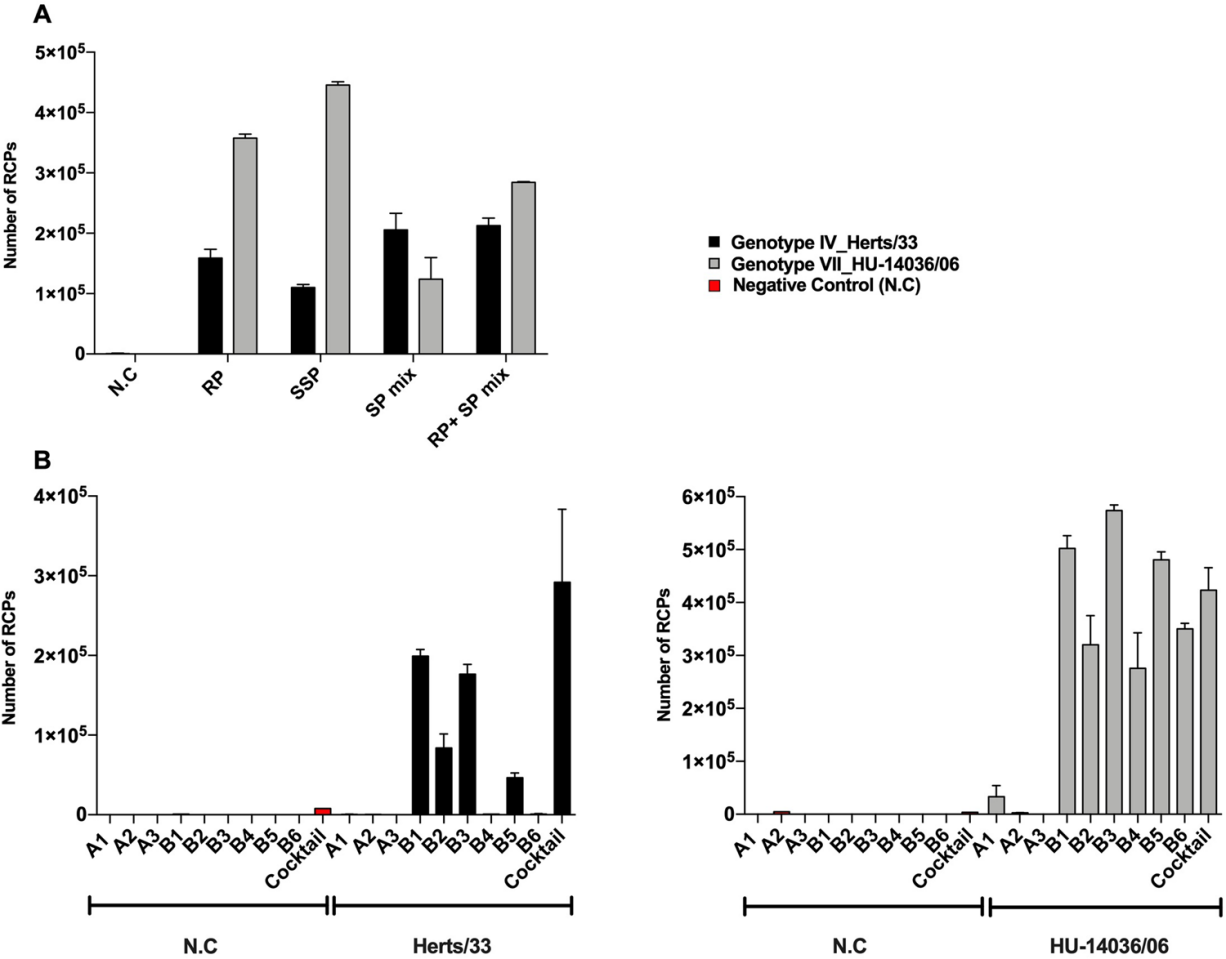
(**A**) Optimization of RT parameters for the choice of primers (RP: Random Primers, SSP: Single Specific Primers, SP mix: mixture of individual specific primers) for Herts/33 and HU-14036/06; 1:10 dilution of cDNA synthesized using SSP B1 and B3, respectively, and amplified using PLP B1 and B3 were used. (**B**) Performance of individual and cocktail PLPs for C2CA validation using two chosen strains, along with their respective negative controls (water instead of template); cDNA dilution of 1:20, synthesized using RP.

Since the use of multiple probes in a relatively close proximity could hinder the performance of the RCA, RT conditions were first optimized. For this random primers, specific primers and combination of both were tested on two genotypes (NDV IV Herts/33 and NDV VII HU_14036/06) and, assessed by determining cDNA synthesis (SI, ST3) and RCP yields (Fig. 3A). All primer conditions resulted in efficient detection of the chosen genotypes, however subsequent experiments were carried out using only random primers since it simplifies assay design and reduces the risk of assay failure due to emerging sequence variation in the binding site for a specific primer. The synthesized cDNA from different genotypes was tested for serial dilutions by RT-C2CA (SI, Fig. S3).

Prior to testing the designed PLPs on virus isolates, the performance and specificity of the individual PLPs was first confirmed with their respective synthetic targets (SI, Figs S4, S5). Next, we assessed the performance of individual PLPs on these two genotypes. Individual and cocktail PLPs were able to detect these NDV strains with different efficiencies (Fig. 3B), thus underlining the advantage of using a cocktail of PLPs for the detection of NDV. Genotype IV could not be detected by PLP B4 and B6 and neither by the A PLPs. In contrast, genotype VII was picked up by all 6 B-PLPs and even the A1 PLP. The PLP cocktail has also been tested with a combination of random and specific primers, where the PLP cocktail could detect both strains of NDV (SI, Fig. S6). Thus, these results underline the advantage of using a cocktail of PLPs for the detection of NDV as both genotypes were detected with it.

### Analytical performance on virus isolates

**Figure 4:**
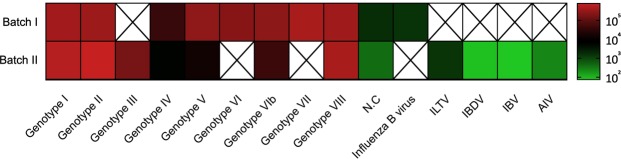
Heat map showing the detection of NDV isolates in two experimental batches obtained from two different sources. The specificity controls with other related viruses are included alongside. For Batch-I, 1:50 dilution of cDNA, and for Batch-II, a final copy number of 10^4^ was used for the samples. The RCP counts obtained by C2CA are illustrated as intensity difference in the heat map (*n* = 2). X symbol denotes no performance of experiments with these conditions.

The analytical performance of the designed PLP cocktail was assessed on virus isolates corresponding to diverse NDV genotypes within the Class II, obtained in two different batches (Batch I and II) provided by SVA, Sweden and NEBIH, Hungary independently (SI, ST4). These genotypes used for validation, including only Class II NDV, were selected based on their high clinical prevalence and availability. Class I NDV, except for one case detected in the 1990s, generally causes symptomless infections^31^. We were able to detect the 7 NDV strains included in batch I, corresponding to genotypes I, II, IV, V, VI, VII and VIII, using the NDV-PLP cocktail (Fig. 4). This was also the case for the second batch of samples, where 6 strains could be detected (Fig. 4). In addition, we confirmed the specificity of our assay with negative and other specificity controls including non-NDV avian viruses (covering both RNA and DNA, positive- and negative-sense strands, single- and double-stranded), such as ILTV, IBDV, IBV and AIV. All these reflect on a robust assay design and performance of the PLP cocktail on isolated strain samples.

Likewise, we tested an avian virus closely related to NDV from a recent outbreak in Hungary, namely Pigeon paramyxovirus type 1 (PPMV-1) (HU-202/16) which is clustered into genotype VIb^32,33^ and shows around 95% similarity to L-gene of NDV^34^. We were able to detect this strain with our NDV-PLP cocktail, thus underlining the versatility and extendable use for very closely related and newly emerging strains (Fig. 4) (SI, Fig. S3B). Specificity controls run for this set of samples included a water negative control and human Influenza B virus, both yielding RCP counts within the background level.

**Figure 5:**
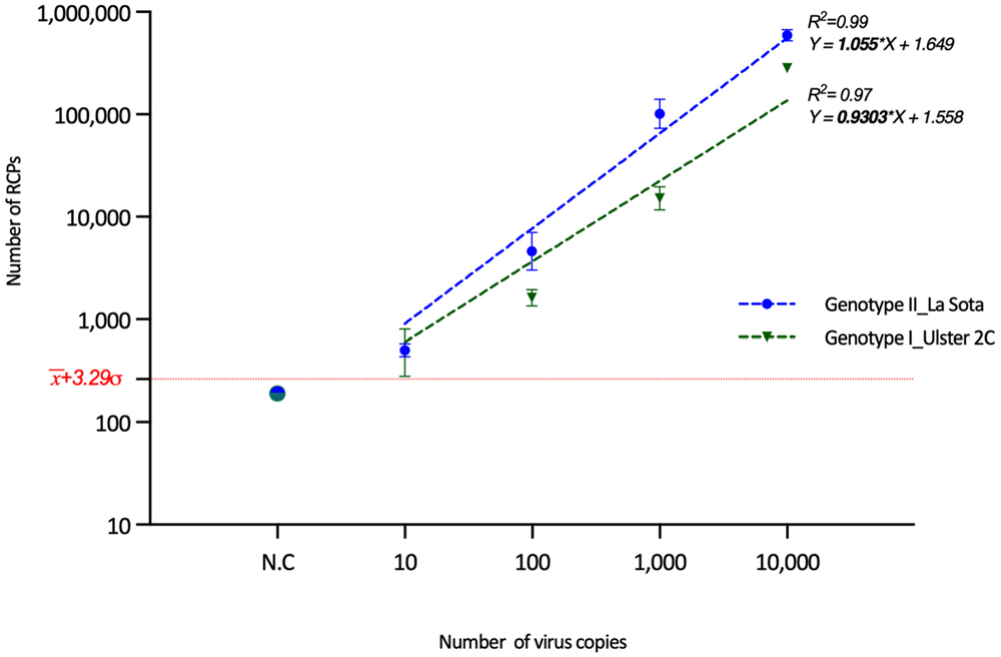
Estimation of limit of detection (LoD) for two chosen strains using C2CA assay and ASMD read-out. LoD was estimated from the linear fit (obtained from the log values) and the background (calculated as 257 RCPs, the mean negative control ($\bar{{\rm{x}}}$) + 3 st. dev. (σ) from the mean), represented in the figure.

Next, the sensitivity of the developed assay was tested by estimating the limit of detection (LoD)^35^ using serial dilutions with the strains Ulster and LaSota (Fig. 5). The estimated LoD was calculated from the linear fit of the experimental data resulting in 5 and 8 copies of NDV RNA for LaSota and Ulster 2C, respectively. The detection of such a low copy number using the developed assay are important at early stages of infection for improved diagnostics in poultry and other industries.

### Multiplexed detection of avian RNA viruses

**Figure 6:**
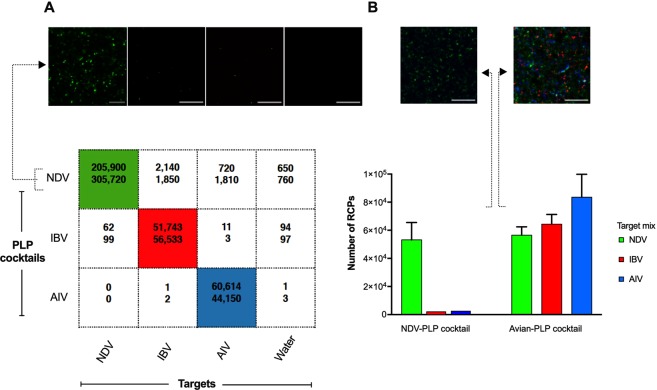
Multiplexing of hypervariable RNA avian viruses using microfluidic membrane enrichment. (**A**) A table showing the RCP values obtained from membrane enrichment readout (n = 2) for the detection of isolated RNA from field strains using respective PLP cocktail for specificity testing with their matching and non-matching targets, along with water negative control. Inset shows images corresponding to the performance of NDV PLPs on various individual targets. Scale bar = 20 μm. (**B**) The multiplexing performance of cocktail PLPs to detect the mixture of three chosen targets is represented using only NDV-specific cocktail PLPs as well as complete cocktail set; specific fluorophores tagged for each of the viruses were used: NDV – Cy3 (green); IBV – Cy5 (red); AIV – Cy7 (blue). Inset shows merged 3-channel images corresponding to each condition. Error bars are derived from triplicate measurements of membrane-enriched samples for complete cocktail, and duplicate measurements for NDV cocktail experiments.

In order to take full advantage of the multiplex capability of PLPs, we extended our design to be able to detect other related poultry viruses. Therefore, we designed additional PLP cocktails to detect IBV and AIV strains and adapted a microfluidic RCP enrichment cartridge for digital quantification. Simultaneous detection of different viruses in the same mixture of sample in a single reaction was enabled by using unique fluorophore-tagged detection oligonucleotides. The specificity of NDV-, IBV- and AIV-PLP cocktails was tested using them independently and as a mix (Fig. 6A). The designed PLP cocktails (NDV-PLP cocktail) were confirmed to be specific up to 100% for their matching targets while very low RCP counts were resulted from their non-matching targets. This was also the case when using all cocktails in single a mix (Avian-PLP cocktail), where the specificity was preserved despite of the high complexity and degeneracy level of the designed probes (Fig. 6B).

## Discussion

The biggest challenge in disease control of single-stranded RNA viruses is due to their high mutation rate that leads to ever evolving new strains and possible epidemics. Conventional assays for molecular detection of viruses depend on either serological or nucleic acid–based methods. For the latter, qPCR has been the gold standard due to the high sensitivity and real-time monitoring of the assay. However, quantitative PCR (qPCR) assays are prone to false negative results caused by new genetic strain variation^36^. Moreover, the sensitivity of qPCR assays for NDV detection are usually in the range of few hundreds copies of RNA^37,38^ for variant strains, thus requiring methods for enhanced sensitivity. Isothermal assays such as LAMP (Loop-mediated isothermal amplification), RPA (Recombinase polymerase amplification), and SDA (Strand displacement amplification) are also seemingly popular in viral diagnostics due to their efficiency and sensitivity. In this context, PLPs in combination with RCA have demonstrated to be a highly specific and robust molecular detection approach^23^. The current study successfully exploited a novel PLP design strategy in conjunction with a robust RCA-based assay design to perform molecular detection of highly variable emerging strains of viruses using NDV as a model. Different from previous studies or other reported approaches that usually target single highly conserved regions for identification of specific target sites^16,17^, we implemented a strategic probe design that rather takes into account the variations by targeting several sites of the genome and inclusion of degenerated bases (Fig. 2D). Moreover, these wobbles were included taking into account the fingerprint of the Tth DNA ligase, thus no mismatch was allowed until 5 bp from the ligation site. The L-gene is the largest of all NDV genes, thus offering a possibility to accommodate many PLPs and increase the coverage. By this, we aimed to not only detect the existing strains, but also emerging ones while preserving the specificity towards NDV viruses. We thus designed 9 PLPs based on an alignment that included representative sequences from the L-gene of NDV, including strains belonging to Class I and II genotypes (Fig. 2). We confirmed the advantages of the current design by successfully detecting strains such as HK-99, PE-8, BG-31 and IT-147 for which we did not possess the sequence information for the L-gene, but only F-gene. Furthermore, we confirmed this by successfully detecting a pigeon virus strain, which was isolated from a recent outbreak in Hungary, and can be considered analogous to emerging NDV strains (Fig. 4). Therefore, it is likely that our design will also detect new emerging strains, and as per need, this panel can be extended to cover emerging strains with new alterations in the sequences.

Additionally, we observed the advantage of using this cocktail when for instance, individual PLPs B4 and B6 could not detect Herts/33, but could detect HU_14036/06 (Fig. 3B) that belong to the same subgroup. This could probably be attributed to variations in the positioning of mutations respective to the ligation site that could affect the efficiency. However, when using the cocktail we were able to detect them both with high efficiency, showing the adaptive feature for mutation-tolerant detection in multiple sites. This also underlines the differences noticed by the performance of PLPs targeting the subgroup A (A1 PLP from Fig. 3B), and its selective ability for detecting sequences of strains haplotyped for the subgroup B (HU_14036/06). The increase in background signal when using the PLP cocktail can be attributed to the cumulative backgrounds from individual PLPs, and this can be addressed by decreasing the concentration of PLPs during the ligation step^20^. Also a careful selection must be performed to remove PLPs that alter the overall background performance of the assay.

Our assay could detect as low as ten copies of the virus RNA, reflecting on the high sensitivity offered by PLPs. We use a digital read-out system for our measurements, where one signal corresponds to one product generated from a single reacted PLP. Thus, the final obtained signal levels could be directly related to the concentration of the pathogen present in the analysed sample. The advantage of RCA digital measurement is that the amplicons cannot dilute or affect each other, but rather enables simple counting of generated molecules, thus reducing amplification bias and providing means for improved sensitivity and precise quantification^24^.

Multiplexing is a key element in addressing parallel diagnostics of relevant strains as well as related viruses for timely and efficient detection. Multiplex NDV detection have been mostly based on RT-PCR, for instance in NDV-pathotyping for discriminating lentogenic and meso/velogenic variants^13,39^. In the current study, we extended the multiplexing towards differential wide detection of relevant viruses. This is achieved by designing additional PLP cocktails and adapting the RCP enrichment method. We demonstrated the high multiplexing ability of our approach preserving 100% specificity, thus bringing the method closer to the actual demands for precise identification of virus infections down to strain level in the field. As an extension, other read-out platforms supporting multiplexing, Luminex for instance, can be combined with the developed assay by suitable modifications like introduction of appropriate barcodes in the PLP backbone for wide detection of many viruses^40^. Interestingly, this enrichment digital read-out strategy can be exploited to reduced assay complexity, and enhance the sensitivity for detecting low pathogen titers^26^. Combining the advantages offered by these various aspects of the assay, the current set-up could be effectively adapted and extended towards simultaneous detection of a wide panel of viruses, including clinically significant ones like Influenza virus, HIV and Hepatitis C virus that are rapidly mutating and thus demand immediate diagnostic attention.

## Supplementary information

A novel mutation tolerant padlock probe design for multiplexed detection of hypervariable RNA viruses
