# Supplementary material for: A novel mutation tolerant padlock probe design for multiplexed detection of hypervariable RNA viruses
Source: Sci Rep. 2019 Feb 27;9:2872. doi: 10.1038/s41598-019-39854-3 (PMC6393471; doi:10.1038/s41598-019-39854-3)
Supplement: Supplementary file 1 — A novel mutation tolerant padlock probe design for multiplexed detection of hypervariable RNA viruses [file 41598_2019_39854_MOESM1_ESM.pdf]

A novel mutation tolerant padlock probe design for multiplexed detection of hypervariable RNA viruses

Sibel Ciftci[1], Felix Neumann[1], Iván Hernández-Neuta[1], Mikhayil Hakhverdyan[2], Ádám Bálint[3], David Herthnek[1], Narayanan Madaboosi[1*], Mats Nilsson[1*]

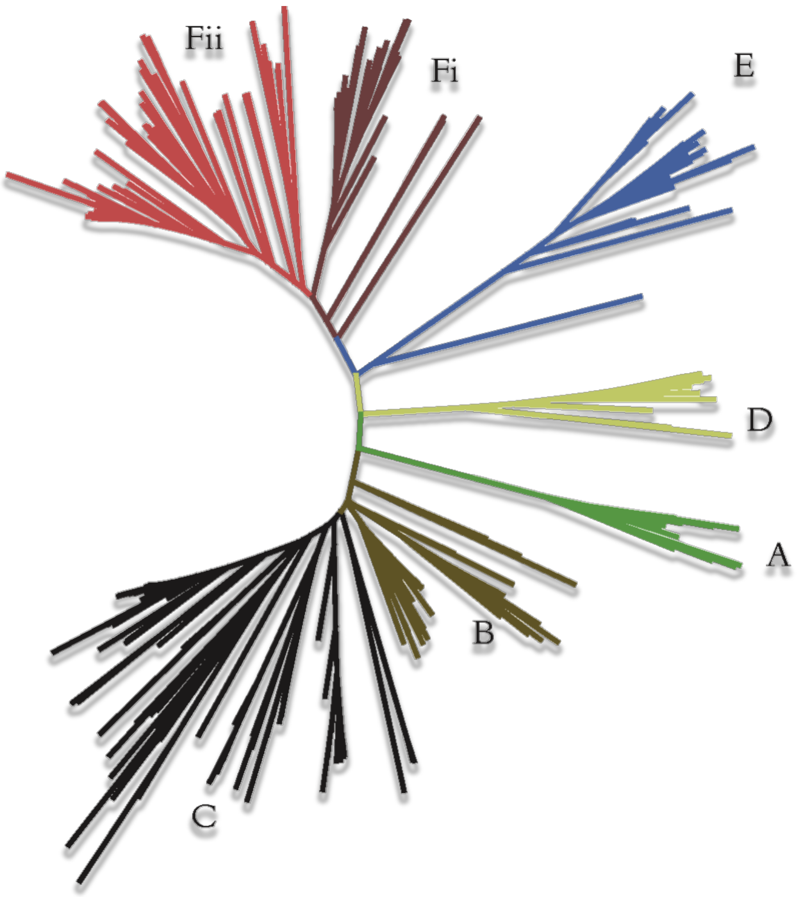

SI, Fig. S1: Phylogenetic tree construction over N-gene of IBV, and its groups (A, B, C, D, E, F_i, F_{ii}).

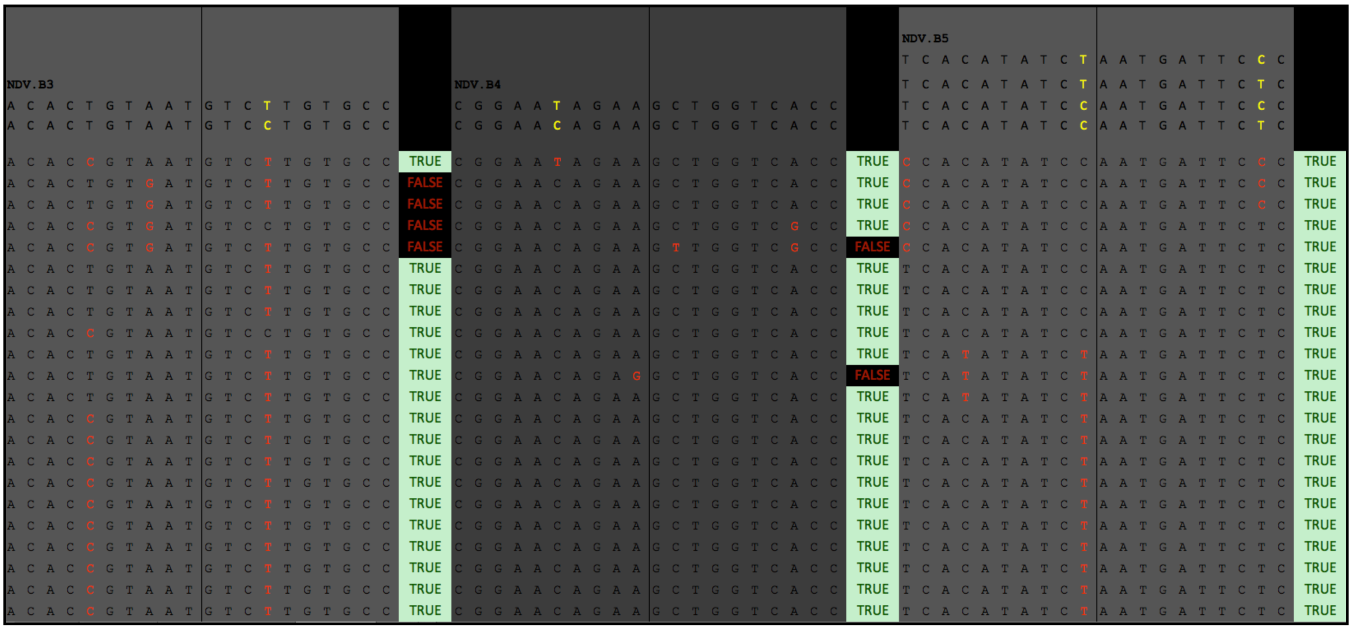

SI, Fig. S2: Output of extracted sequences obtained from Geneious 'Alignment View', showing the position of mismatches in individual sequences relative to the consensus. The position of yellow coloured bases in the consensus will be accommodated by Wobbles during PLP design. Mismatches to any of the consensus sequences is represented in red colour. 'True' or 'False' scoring is based on the positioning of mismatches (5 bp) in relation to the ligation site.

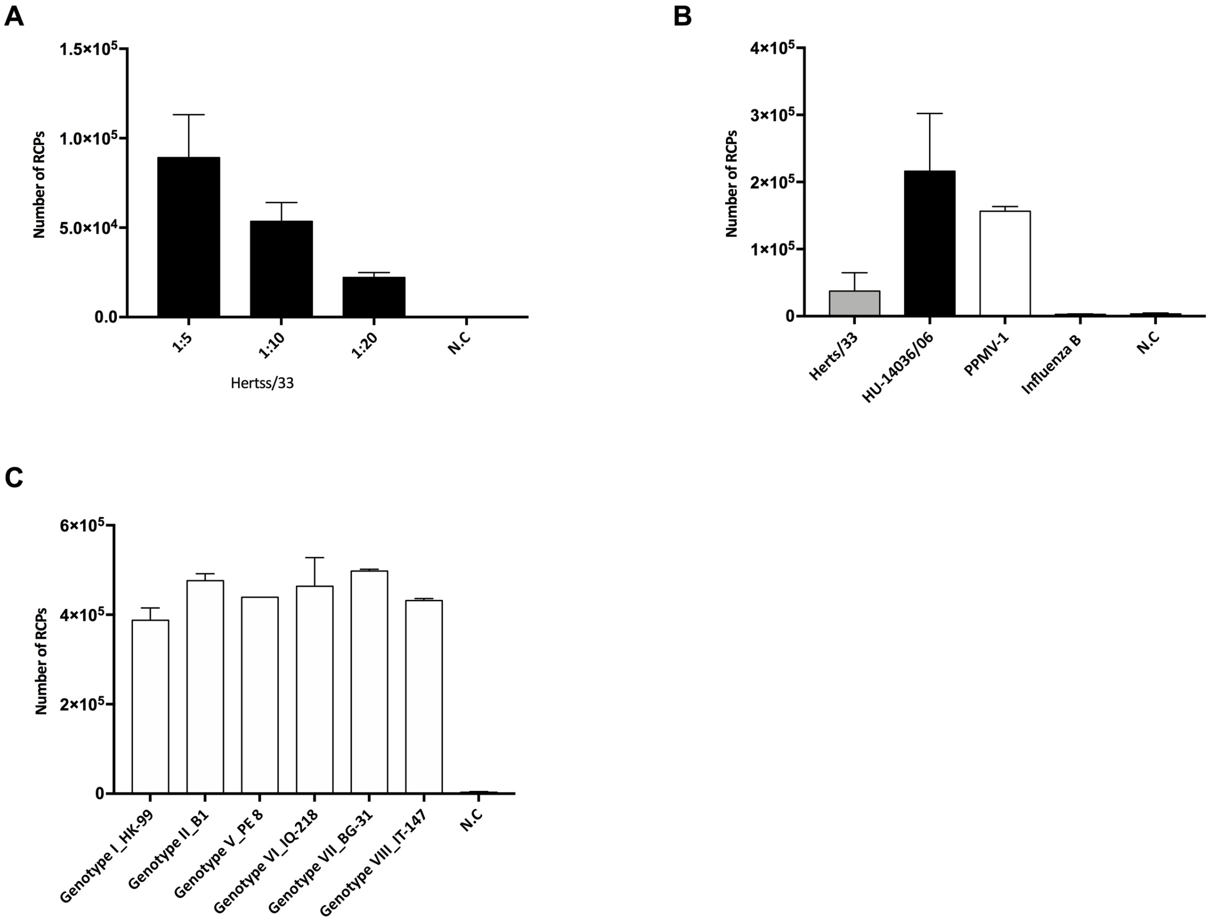

SI, Fig. S3: Optimization of RT-C2CA experiments. **A.** Different concentrations of cDNA from Herts/33 synthesized using SP (B1) and amplified using PLP (B1). **B.** 1:40 dilution of cDNA from different strains (along with Influenza B virus as specificity control), synthesized using RP and amplified using cocktail PLP. **C.** 1:10 dilution of cDNA from different strains, synthesized and amplified as in **B**.

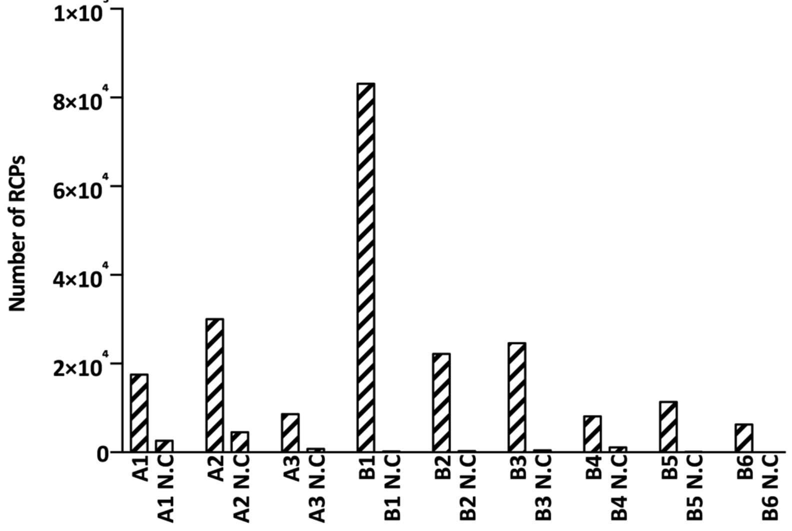

SI, Fig. S4: PLP validation by RT-C2CA assay on their respected synthetic targets (100 fM) along with their negative controls (water instead of synthetic target).

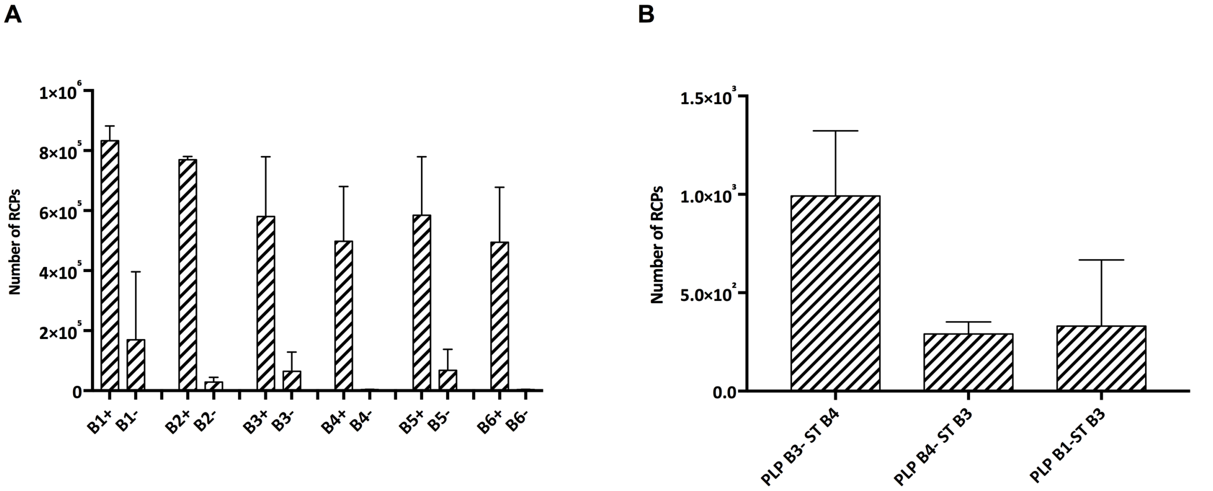

SI, Fig. S5: RT-C2CA assay performed using synthetic targets (ST) at a concentration of 100 fM and cocktail PLP for the specific performance of the PLPs used. **A.** Use of specific PLPs, alongside showing the results after the elimination of the respective PLP from the cocktail (e.g., B1+ uses cocktail PLP for detecting B1 synthetic target; B1- has elimination of B1 PLP from the cocktail). **B.** Cross-reactivity of PLPs on synthetic targets.

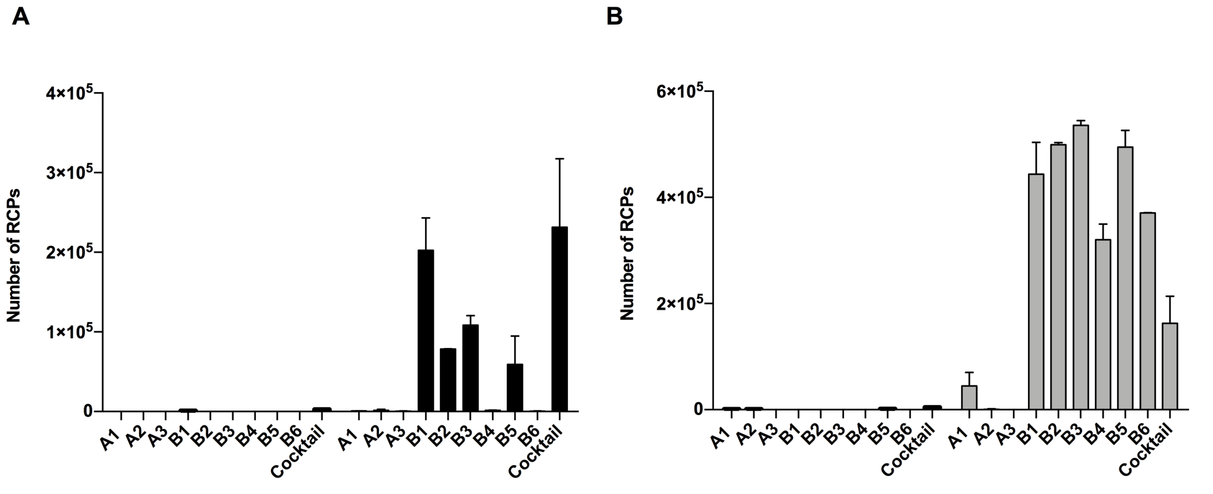

SI, Fig. S6 Performance of individual and cocktail PLPs for C2CA validation using **A.** Herts/33 and **B.** HU-14036/06, along with their respective negative controls (water instead of template). cDNA (dilution of 1:20) synthesized by SP (B1 for Herts/33 and B3 for HU-14036/06) + RP was used.

SI, ST1. Synthetic targets used in this study

Name	5´modification	Sequence
NDV.A1	Biotin	TGCGAGAGGGGGWATTGAGGGGCTTTGCCAGAA
NDV.A2	Biotin	AAGACAGAGAGACTATCAGRTCAGATACTTTCTTCATCTA
NDV.A3	Biotin	TATATCGAGTGTCTCCATATGTCCATATATCAAATGACT
NDV.B1	Biotin	CAGGGAAATGGGGTCATTCARGATAGCATATCCTT
NDV.B2	Biotin	TGCAAAAGTATTGTCTTAAYTGGAGATATCAGACAGTCAA
NDV.B3	Biotin	GTGAAAACACTGTAATGTCYTGTGCCAACATTGCATC
NDV.B4	Biotin	AGATTATGCCCGGAAYAGAAGCTGGTCACCTTTGACAGG
NDV.B5	Biotin	CACCTTACATTCACATATCYAATGATTCYCAAAGGCTATT
NDV.B6	Biotin	TGACTCGTGCTCAACAAAAATTSTAYATGAAAACCATAGG

SI, ST2. Table of oligonucleotides used for other avian viruses

Name	5´modification	Sequence
ILTV_PLP.1	Phosphate	GAAGGATTGATTTCTGGAACGTGTATGCAGCTCCTCAGTAATAGTGTCTTACCGCCAACGTGAGCATCGTATAATAGCGTCTGGTCGATT
ILTV_PLP.2	Phosphate	CGACGTAAAGAAGTACAACGTGTATGCAGCTCCTCAGTAATAGTGTCTTACCGCCAACGTGAGCATCGTATACTCCGAATGGCCCGT
IBDV_PLP.1	Phosphate	GTCAAATTGTAGGTCGAGGTGTGTATGCAGCTCCTCAGTAATAGTGTCTTACCCTGCGAGTGGTCACGAATACCCTGTGTCCCCCACA
IBDV_PLP.2	Phosphate	CCACAGTCAAATTGTAGGTGTGTATGCAGCTCCTCAGTAATAGTGTCTTACCTCCGCACTGGTCAGGTATACCTGACCCTGTGTCCC
IBDV_PLP.3	Phosphate	CACTCAGTATCAATTTTGTGGTGTATGCAGCTCCTCAGTAATAGTGTCTTACCTCCGCACTGGTCAGGTATAGCCAAGACGGTCCCTCT
IBDV_PLP.4	Phosphate	TTCCTGACGCGGCTCGGTGTATGCAGCTCCTCAGTAATAGTGTCTTACCTCCGCACTGGTCAGGTATACTGAGGCAGCTCTTGCTT
IBDV_PLP5	Phosphate	TCTCTGCATGCACGTAGTCGTGTATGCAGCTCCTCAGTAATAGTGTCTTACCTCCGCACTGGTCAGGTATATGATGCCAACCGGCTCT
IBV_PLP.1	Phosphate	CACTTGGTGAAAATGAACTTGTGTATGCAGCTCCTCAGTAATAGTGTCTTACCTGTCCCGTGTGCTGAGATAGATTAATTGGGGTGATTCAG
IBV_PLP.2	Phosphate	AAAGCATTGACCTCAGATGAGTGTATGCAGCTCCTCAGTAATAGTGTCTTACCTGTCCCGTGTGCTGAGATAGCAGGATGATGAAGTRGAT
IBV_PLP.3	Phosphate	GTTGCTGCAAAGGGTGCTGTGTATGCAGCTCCTCAGTAATAGTGTCTTACCTGTCCCGTGTGCTGAGATACAAGATGGTATAGTGTGG
IBV_PLP.4	Phosphate	AATGAGGARGGTATTAAGGGTGTATGCAGCTCCTCAGTAATAGTGTCTTACCTGTCCCGTGTGCTGAGATATTTGGTGATGACAAGATG
IBV_PLP.5	Phosphate	AATGAGGARGGTATTAAGGATGGTGTATGCAGCTCCTCAGTAATAGTGTCTTACCTGTCCCGTGTGCTGAGATAAATTTTGGTGATGACAAGATG
IBV_PLP.6	Phosphate	GTGTGGGTTTCTGCAAAGGGTGTATGCAGCTCCTCAGTAATAGTGTCTTACCTGTCCCGTGTGCTGAGATATAGCCAAGATGGTATA
IBV_PLP.7	Phosphate	GTGTGGGTTGCTGCAAAGGGTGTATGCAGCTCCTCAGTAATAGTGTCTTACCTGTCCCGTGTGCTGAGATATAGCCARGATGGTATA
IBV_PLP.8	Phosphate	TTTTGGAAGTAGAGTGACGGTGTATGCAGCTCCTCAGTAATAGTGTCTTACCTGTCCCGTGTGCTGAGATATAGTAGCCATGCTTGTCT
IBV_PLP.9	Phosphate	CGTACTAAAGGTAAGGAGGGTGTATGCAGCTCCTCAGTAATAGTGTCTTACTTCTGTCCCGTGTGCTGAGATATTGATAAAGTTTTTGGCCCT
IBV_PLP.10	Phosphate	CCTGATAATGAAAATCTTAAAGTGTATGCAGCTCCTCAGTAATAGTGTCTTACCTGTCCCGTGTGCTGAGATAGTTTGAAGGTAGTGGTGTT
IBV_PLP.11	Phosphate	GACCTGAATTGGGGTGATTGTGTATGCAGCTCCTCAGTAATAGTGTCTTACCTGTCCCGTGTGCTGAGATAAACAGGACCAGCCGCT
AIV_PLP.1	Phosphate	CRCTGGGCACGGTGAGTGTATGCAGCTCCTCAGTAATAGTGTCTTACAAATCAGTCATACGAGCGCCGCTGCAGTCCYCGYT

SI, ST3. Concentrations of cDNA samples used for assay optimization.

Primer and cDNA conditions	**RP**	**SSP**	**SP mix**	**RP + SP mix**
**Genotype IV_ Herts/33**	3.8 ng/µL	0.800 ng/µL	3.16 ng/µL	4.64 ng/µL
**Genotype VII_HU-14036/06**	7.00 ng/µL	4.60 ng/µL	8.20 ng/µL	12.3 ng/µL

SI, ST4. List and details of viral strains used in the study.

	Strain Name	Genotype /virus	NCBI	Virulence*	cDNA (ng/ µl)	Copy number/ µl	Provider
BATCH-I	HK-99	I	EU604269	L	1.87		SVA (Sweden)
	B1	II	AF309418	L	4.40		
	Herts/33	IV	FJ687487	V	3.8		
	PE-8	V	EU604263	V	1.62		
	IQ-218	VI	EU604254	M	3.70		
	BG-31	VII	EU604250	V	5.16		
	IT-147	VIII	EU604262	V	3.18		
BATCH-II	Ulster 2C	I	AY562991	L		10^4	NEBIH (Hungary)
	La Sota	II	AJ629062	L		10^5	
	Ph/80	III	FJ687488	M		10^4	
	Herts/33	IV	FJ687487	V		10^5	
	HU-206/81	V	-	V		10^5	
	HU-202/16**	VI	-	M		10^5	
	HU-14036/06	VII	-	V		10^4	
	IT-147/94	VIII	EU604262	V		10^4	
Non-NDV strains	Cevac LT L	ILTV	-			10^4	NEBIH (Hungary)
	Hipragumboro-CH/80	IBDV	-			10^6	
	11518/10	IBV	-			10^7	
	A/duck/Hungary/54494/16 (H5N8)	AIV	-			10^6	
	B/Stockholm/5/2014 (Victoria)	Influenza B	-			$2x10^4$	Karolinska institute (Sweden)

* L: Lentogenic M: Mesogenic V: Velogenic
** PPMV-1: Pigeon paramyxovirus type 1 (https://www.ncbi.nlm.nih.gov/pmc/articles/PMC2566108/)
